# Heteroplasmic mitochondrial genomes of a *Raillietina* tapeworm in wild Pangolin

**DOI:** 10.1186/s13071-022-05301-y

**Published:** 2022-06-13

**Authors:** Merga Daba Tuli, Hongyi Li, Xi Pan, Song Li, Junqiong Zhai, Yajiang Wu, Wu Chen, Wanyi Huang, Yaoyu Feng, Lihua Xiao, Dongjuan Yuan

**Affiliations:** 1 Guangdong Laboratory for Lingnan Modern Agriculture, Center for Emerging and Zoonotic Diseases, College of Veterinary Medicine, South China Agricultural University, Guangzhou, 510642 China; 2 Guangzhou Zoo and Guangzhou Wildlife Research Center, Guangzhou, 510070 China

**Keywords:** Mitochondrial DNA heteroplasmy, Phylogenetic analysis, *Raillietina* species, Tapeworm

## Abstract

**Background:**

*Raillietina* species belong to the family Davaineidae, which parasitizes in a wide variety of mammals and birds, causing stunted growth, lethargy, emaciation, and digestive tract obstruction. However, only a limited number of *Raillietina* species have been identified in wild animals.

**Methods:**

We analyzed and annotated the complete mitochondrial (mt) genome of a worm from the intestine of a wild pangolin using Illumina sequencing of whole genomic DNA.

**Results:**

These findings showed the presence of two mtDNA sequences in *Raillietina* sp., designated as mt1 and mt2, with the lengths of 14,331 bp and 14,341 bp, respectively. Both the mts genomes of *Raillietina* sp. comprised 36 genes, containing 12 protein-coding genes (PCGs), 2 ribosomal RNAs, and 22 transfer RNAs. Gene arrangements of both mt genomes of *Raillietina* sp. were similar to those of most flatworms, except for taeniids, which shift positions between tRNAL1 and tRNAS2 genes. Twenty of 22 tRNA secondary structures of *Raillietina* sp. had a typical cloverleaf structure similar to *Raillietina tetragona*. Sequence differences between the mt1 and mt2 genomes were 4.4%, and this difference arises from the mtDNA heteroplasmic mutations. Moreover, heteroplasmic mtDNA mutations were detected in PCGs, tRNAs, rRNAs, NCRs, and intergenes, but the highest proportion of heteroplasmy of 79.0% was detected in PCGs, indicating the occurrence of mtDNA heteroplasmy in *Raillietina* sp. To our knowledge, this is the first report of mtDNA heteroplasmy in tapeworm parasites. Phylogenetic analyses of 18S rRNA, *ITS2*, and 12 PCG sequences demonstrated that the worm was clustered with other *Raillietina* species in the Davaneidae family.

**Conclusions:**

We found a novel *Raillietina* species in wild pangolin with the existence of mitochondrial DNA heteroplasmy. Thus, these findings provide insights into the heterogeneity of the mt genome in parasitic cestodes, and mt genome data contributes to the understanding of pangolin-parasitic cestodes in terms of their molecular biology, epidemiology, diagnosis, and taxonomy.

**Graphical Abstract:**

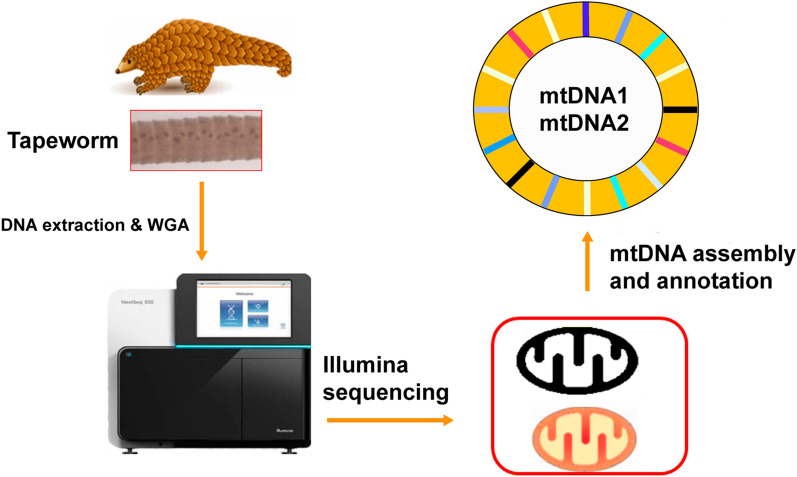

**Supplementary Information:**

The online version contains supplementary material available at 10.1186/s13071-022-05301-y.

## Background

Cestodes are obligate parasites and widely parasitize in domestic and wild animals [1]. Cyclophyllidea is one of the orders in the cestode with the most diverse species, and it includes 3100 species from 15 families [2]. Genus *Raillietina* belongs to the family Davaineidae in the order of Cyclophyllidea. *Raillietina* species parasitize in the small intestine of the hosts, causing stunted growth, lethargy, emaciation, and digestive tract obstruction [3]. Infections occur commonly via ingestion of intermediate hosts, beetles, ants, small mini-wasps, termites, and cockroaches containing infective cysticercoid [4].

Morphological analysis has been used to identify minor differences in scolex shape and size, rostellum hooks (unarmed or armed), gravid proglottids, and suckers of Cyclophyllidea species [5]. Although morphological investigations have been widely employed in species taxonomy, there are some limitations in accuracy, duration, and labor intensiveness. Furthermore, this method is also inadequate for identifying inter-related species in the order Cyclophyllidea. Currently, accurate species identification plays an essential role in the diagnosis, prevention, and control strategies of cestodes. Thus, molecular markers of nuclear ribosomal DNA (internal transcribed spacer/ITS2) and mitochondrial DNA (nicotinamide adenine dinucleotide/NAD1) have been extensively used for the identification of *Raillietina* species [3]. Moreover, the mtDNAs of cestode species have been widely characterized and used for the study of molecular epidemiology, population genetics, diagnosis, and resolving phylogenetic relationships because of its mt genomic features of maternal inheritance and absence of recombination [6, 7].

Pangolins are highly trafficked mammals globally and are distributed in tropical Asia and sub-Saharan Africa. Ants and termites are the main food sources of pangolins [8]. Most Davaineidae parasites require ants and termites as intermediate hosts to complete their life cycle [9]. Thus, pangolins are more likely to be infected with cestode species because of the ingestion of insects containing *Raillietina* cysticercoid. However, the identification of cestode species in wild pangolins is highly insufficient. In addition, there is a scarcity of mt genome data of *Raillietina* species in domestic and wild animals. To date, only one complete mt genome of *Raillietina tetragona* has been sequenced and characterized from chicken [10]. Moreover, there is still a lack of detailed investigation of the mt genome of *Raillietina* species. Thus, the objective of this study was to characterize the mitochondria of a novel *Raillietina* sp. and to infer the phylogenetic relationship with related species based on protein coding genes (PCGs) sequences.

## Methods

### Parasite collection

Pangolins were confiscated from poachers and rescued at the Guangzhou Zoo, Guangdong Province, P.R. China. Of these, one pangolin had severe trauma and complicated infection of the forelimb and ultimately died as a result of infection. In total, six adult worms were collected at necropsy from the small intestine of a pangolin. Adult parasites were thoroughly washed with a phosphate-buffered saline and were preserved with 75% ethanol for further identification and were kept at – 20 °C till use.

### DNA extraction and whole-genome amplification (WGA)

Total genomic DNA was extracted from the posterior part of a segment of a single worm using the Wizard® SV Genomic DNA Purification System (Promega, Madison, WI, USA) according to the manufacturer’s instructions and stored at – 20 °C until use. Whole genome amplification was conducted using REPLI-g® Midi Kits (Qiagen^®^, Germany). The amplified DNA was sequenced by an Illumina Novaseq 6000 using a 150-bp paired-end technique (Illumina, San Diego, CA, USA). Approximately 11.7 Gb of sequence data had a quality score (*Q*-score) ≥ 20.

### PCR amplification and DNA sequencing

The 18S rRNA region of *Raillietina* sp. was amplified by PCR. We used the forward primer SSU-A: 5′-AAAGATTAAGCCATGCATG-3′ and reverse primer SSU-22R: 5′-GCCTGCTGCCTTCCTTGGA-3′ [11]. The total volume of the PCR mixture was 50 µl, containing 10 µl of 5 × PCR buffer, 5 µl of 2 mM dNTPs, 2.5 µl of 10 μM primers, 0.5 µl Phusion DNA polymerase (Thermo Fisher Scientific, Waltham, MA, USA), 28.5 µl dd H_2_O and 1 µl DNA template. The PCR amplification was carried out according to the following program: initial denaturation at 94 °C for 3 min, followed by 40 cycles at 94 °C for the 30 s, 60 °C for 30 s, 72 °C for 60 s, and finally extended at 72 °C for 5 min. The amplified PCR product was checked using 1.5% agarose gel electrophoresis; finally, the PCR fragments were sequenced by Sanger sequencing (Sangon, Shanghai, China). Sequence similarity was detected using BLAST (http://blast.ncbi.nlm.nih.gov/Blast.cgi).

*ITS2* of *Raillietina* sp. was amplified using forward primer 3S (5′-GGTACCGGTGGATCACTCGGCTCGTG-3′) and reverse primer BD2 (5′-TATGCTTAAATTCAGCGGGT-3′) [3]. The PCR reaction was as follows: initial denaturation at 95 °C for 3 min, followed by 35 cycles at 95 °C for 30 s, 58 °C for 30 s, 72 °C for 60 s, and a final extension at 72 °C for 10 min. The amplified PCR product was checked using 1.5% agarose gel electrophoresis. PCR fragments were sequenced by Sanger sequencing (Sangon, Shanghai, China).

### Complete mt genome assembly of worm

A total of 11.7 Gb of raw data was obtained from the *Raillietina* sp. using Illumina sequencing. Sequence data of *Raillietina* sp. were assembled using CLC Workbench software, and two heterogeneities of mitochondrial genomes were obtained, named mt1 and mt2. The mt genome sequences of mt1, mt2, and *R. tetragona* were compared. Consequently, the multiple sequence alignment of mt1, mt2, and *R. tetragona* (MH122786.1) was implemented using the Multiple Sequence Comparison by Log-Expectation (MUSCLE) tool (https://www.ebi.ac.uk/Tools/msa/muscle) and visualized by Jalview.

### PCR amplification and cloning of the NCR region

After CLC assembly of mt1 and mt2, the sequences were found in some regions that were difficult to assemble because of repeat sequences or N-sequences (unidentified sequence). The primers were designed based on 100 bp upstream and downstream of these regions (Additional file 1: Table S1). The fragments were amplified from worm DNA using Dream *Taq* DNA polymerase, and the PCR fragments were checked by 1.5% agarose gel electrophoresis. After purification by the EasyPure^®^ PCR Purification Kit (TransGen Biotech, Beijing, China), the PCR products were cloned into the vector by the *pEASY*^®^-Blunt cloning kit (TransGen Biotech, Beijing, China). The fragments were sequenced by Sanger sequencing (Sangon, Shanghai, China).

### Gene annotation and sequence analysis

The complete mitochondrial genomes of mt1 and mt2 were annotated by mitos software (http://mitos2.bioinf.uni-leipzig.de/index.py) [12]. The PCGs were identified by the NCBI open reading frames finder (ORF Finder) (employing genetic code9, invertebrate mitochondria DNA), and the gene boundary was identified on the mitos web server (http://mitos2.bioinf.uni-leipzig.de/index.py). Each transfer RNA (tRNA) of mt1 and mt2 was predicted, and the tRNA boundary was identified using trnascan-se (http://lowelab.ucsc.edu/tRNAscan-SE/), ARWEN (v1.2.3), and the Mitos web server. To evaluate the copy number of the two mitochondria, the following formula was employed: mitochondrial genome coverage = high-quality data volume/mitochondrial genome length. The high-quality data volume was equal to the number of the reads that mapped to the mitochondrion multiple to their length.

Moreover, secondary structures of non-coding regions (NCR1 and NCR2) were predicted by the mfold program (v5.5.2). The repeat sequences in the NCR of mt1 and mt2 of the *Raillietina* sp. were detected using a tandem repeat finder (v4.09). The PCGs, ribosomal RNA (rRNA), tRNA, NCR1, and NCR2 sequences of mt1 and mt2 were compared with the mtDNA of *R. tetragona* (GenBank accession number: KP057580.1) [10].

### Mitochondrial DNA heteroplasmic identifications

The sequences of mt1 and mt2 of *Raillietina* sp. were compared, and heteroplasmic site distributions between the two mts were also identified using BWA (v0.7.17), SAMtools (v1.7), and BCFtools (v1.8). The percentage of synonymous (dS) and non-synonymous (dN) mutations of PCGs was calculated using DNA sequence polymorphism (Dnasp6) [13].

Codon usage and relative synonymous codon usage (RSCU) for 12 PCGs of mt1, mt2, and *R. tetragona* were computed by DAMBE (v7.3.2) and visualized by R studio. If RSCU > 1, more codons were used than expected, RSCU = 1, no codon bias, and RSCU < 1, fewer codons were used than expected [14].

### Verification of the existence of two mt genomes in *Raillietina* sp. using PCR amplification

To further identify the occurrences of two different mtDNA in *Raillietina* sp., we amplified *nad1* gene fragment. We used two pairs of primers to amplify the *nad1* gene fragment of *Raillietina* sp. mt1 and mt2. The first pair of primers was forward primer JB11 (5′-AGATTCGTAAGGGGCCTAATA-3′ and reverse primer JB12 (5′-ACCACTAACTAATTCACTTTC-3′) [3]. The second pair of primer was designed based on nad1 gene sequence in *Raillietina* sp. mt2. The forward primer sequence was 5′-AGTTTCGTAAGGGACCTAAAA-3′ and reverse primer sequence 5′-TCCACTAACAAATTCACTCTC-3′. The PCR reaction was as follows: initial denaturation at 95 °C for 3 min, followed by 35 cycles DNA denaturation at 95 °C for 30 s, primer annealing at 58 °C for 30 s, extension at 72 °C for 1 min, and a final extension at 72 °C for 10 min. The amplified PCR product was visualized and checked using 1.5% agarose gel.

### Phylogenetic analyses based on 18S rRNA, ITS2, and PCGs

The 18S rRNA sequences of *Raillietina* sp. and 32 cestode species from GenBank were downloaded to construct a phylogenetic tree (Additional file 1: Table S2). *Schistosoma japonicum* (trematode) was used as an outgroup. Then, multiple sequence alignment of all the extracted sequences was done using MAFFT [15]. The maximum likelihood (ML) method was executed to construct the phylogenetic tree using RAxML-ng [16]. The ML tree was made with the HKY + G4 model.

The *ITS2* sequences of the two worms of *Raillietina* sp. and 19 different cestode species were downloaded from the database to construct the phylogenetic tree (Additional file 1: Table S3). The trematode *Schistosoma japonicum* was used as an outgroup. The multiple sequence alignment was performed using MAFFT [15]. We constructed the ML tree with the TPM2uf + I + G4 model using RAxML-ng [17].

The 12 PCG sequences of 18 Cyclophyllidea species, one Pseudophyllidea species, 6 Diphyllobothriidea species, and one trematode species (outgroup) were obtained from GenBank (Additional file 1: Table S4). Twelve PCGs of *Raillietina* sp. mt1, mt2, and 26 other species were used to construct the phylogenetic tree using RAxML-ng [17], maximum likelihood with 1000 bootstrap replicates was used, and ML was made with the TVM + I + G model.

## Results

### Morphological observation of worm

The worm was identified based on its morphological features. The continuous segments (proglottid) and ribbon-like arrangements were observed on the external body. Mature proglottids were found in the posterior part of the segment. A dark continuous stripe structure was also found on the mature proglottid towards the posterior end. One segment of the mature proglottid overlapped with another segment (Additional file 1: Fig. S1).

### Primary identification of worm by 18S rRNA and ITS2

The PCR fragments of 18S rRNA (413 bp) and *ITS2* region (813 bp) were amplified from two worm samples (sample A and sample B). Sequence identity of 18S rRNAs between sample A and sample B was 100%. The BLAST result of the 18S rRNA sequence showed 98.2% nucleotide identity with *Raillietina australis* from GenBank (accession no: AY382311.1). Sequence identity of *ITS2* region between sample A and sample B was 98.9% (Additional file 1: Fig. S2A). Phylogenetic analysis of *ITS2* regions showed that sample A and sample B were clustered with other *Raillietina* species into a branch (Additional file 1: Fig. S2B). This indicated that sample A and sample B were the same tapeworm species from *Raillietina*.

**Fig. 1:**
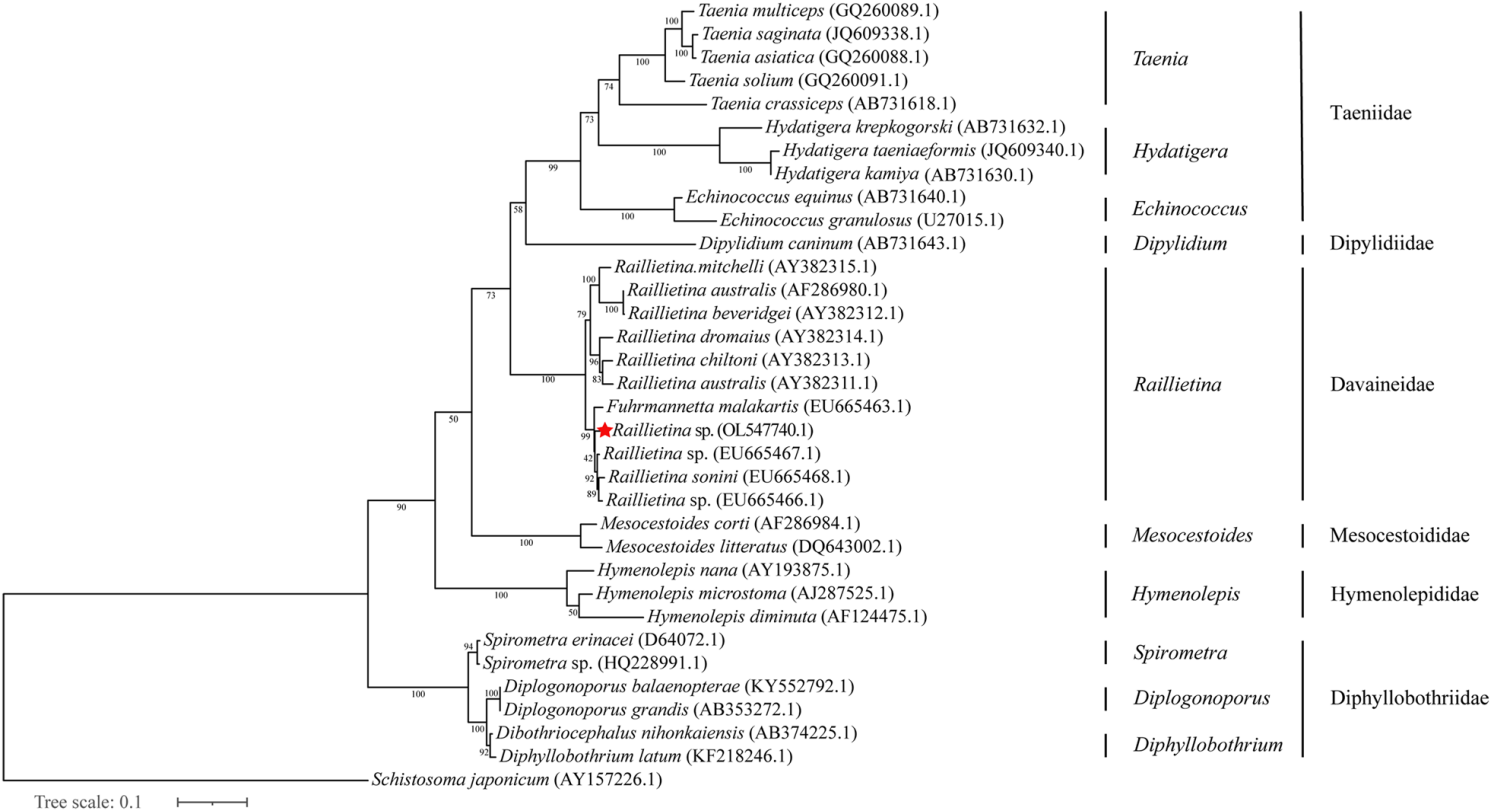
Phylogenetic analysis of *Raillietina* sp. based on 18S rRNA sequence using maximum likelihood (ML) method. *Schistosoma japonicum* was rooted as an outgroup. The scale bar indicates the estimated number of nucleotide substitutions per nucleotide site

The 11.7 Gb of raw data was obtained with 78,238,962 reads from a single *Raillietina* worm using Illumina sequencing. The total 123.2-Mb genome with 78,336 contigs and N50 of 1983 bp was obtained after genome assembly using CLC Workbench. The assembled 18S rRNA sequence of *Raillietina* sp. had been deposited in GenBank with accession number OL547740.1. The BLAST result of the 18S rRNA sequence showed 98.3% nucleotide identity with *Raillietina* sp. from GenBank (accession no. EU665467.1). The 18S rRNA sequences of 32 species in cestode and one species in trematode as an outgroup were used to construct a phylogeny tree (Fig. 1). *Raillietina* sp. was clustered with other species in the family Davaineidae. *Raillietina* sp. was clustered with other *Raillietina* species into a branch, and this species was more closely related to *Fuhrmannetta malakartis* (*Raillietina malakartis*) and *Raillietina* sp. than other species in the family Davaineidae.

### General features of worm mt genome

In the assembled mt genome, the coexistence of two mtDNA sequences was detected and named mt1 and mt2 based on BLAST results. The repeat and the N-sequence/unidentified regions of the mt1 and mt2 were amplified and cloned (Additional file 1: Fig. S3). The length of the repeat sequences was 32 bp (repeated 18 times) and N-sequence/unidentified region was 98 bp. Thus, the complete mt genomes of mt1 and mt2 were 14,331 bp and 14,341 bp, respectively. The sequences of mt1 and mt2 had been deposited in GenBank under accession numbers OL597539.1 and OL597540.1, respectively. After reads mapping and calculation, the mitochondrial coverages of mt1 and mt2 were 3126.18 and 3109.45, respectively, which were very similar. This implies that these two mitochondrial genomes have a similar copy number in *Raillietina* sp. (Additional file 1: Fig. S4).

### The existence of two mt genomes in *Raillietina* sp. by PCR amplification

The PCR fragment of *nad1* (488 bp) was amplified from the *Raillietina* sp. using the primers of JB11 and JB12 [3]. *Nad1* fragment had the same sequences as the *nad1* sequences of mt1. The PCR fragment of *nad1* of *Raillietina* sp. mt2 was amplified by the specific primers based on the *nad1* sequences of *Raillietina* sp. mt2 (Additional file 1: Fig. S5). Alignment result showed that *nad1* from mt1 and mt2 had significantly different sequences. Thus, *nad1* sequences of mt1 and mt2 supported the coexistence of two distinct mitochondrial genomes in *Raillietina* sp.

### Primary comparison of mt1 and mt2 of *Raillietina* sp.

**Fig. 2:**
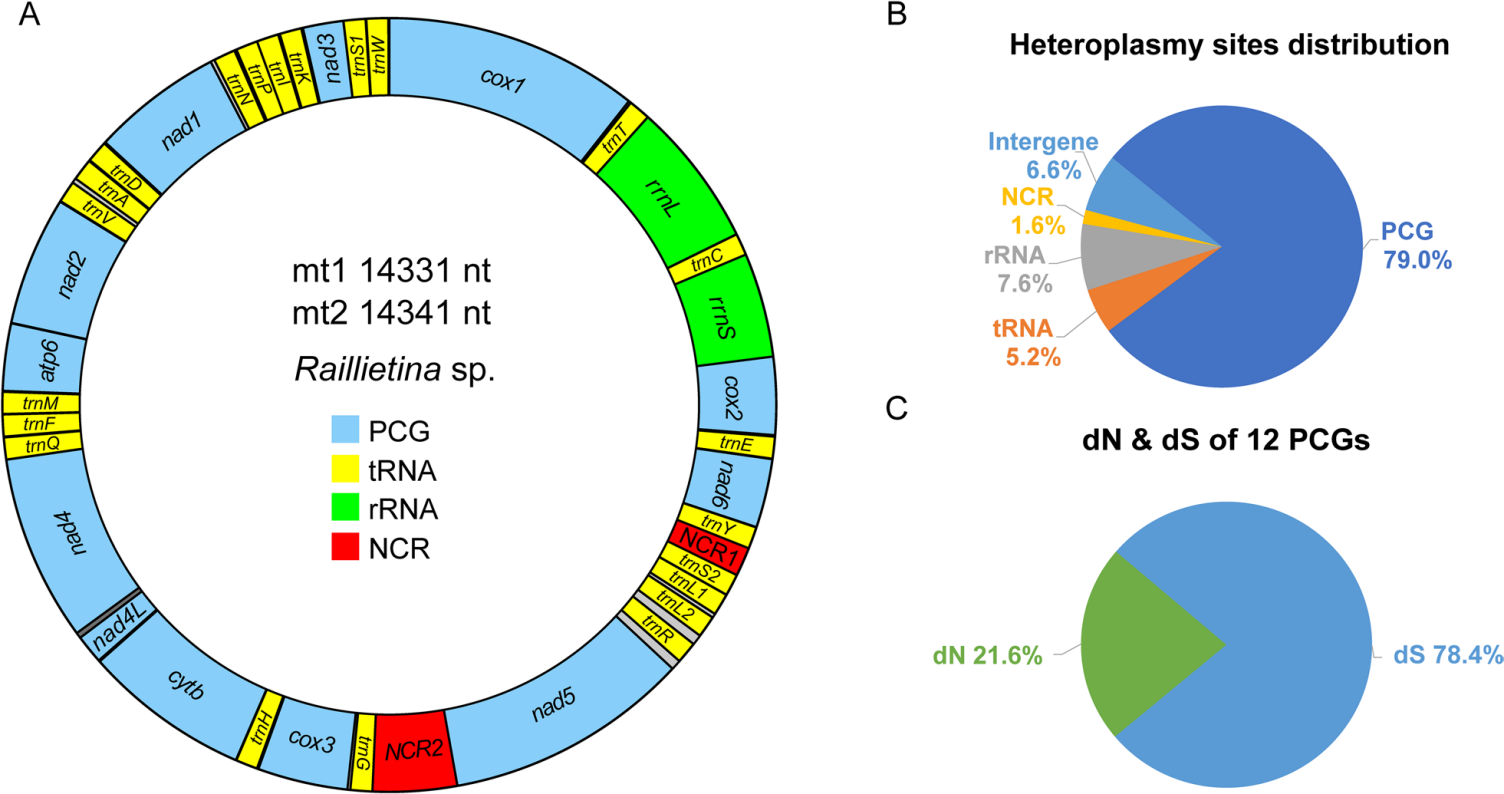
Features of mitochondrial genome mt1 and mt2 of *Raillietina* sp. **A** Mitochondrial genome organization of mt1 and mt2 of *Raillietina* sp. The map presented 12 PCGs, 2 rRNAs, 2 NCRs, and 22 tRNAs denoted by the initial letter of their amino acids. Leucine codon families in mts were identified as L1 and L2, denoted by CUN and UUR, respectively. Serines in mts were identified as S1 and S2, denoted by AGN and UCN, respectively. The inner circle indicates genome size and content. **B** The proportion of heteroplasmic sites distribution in mt1 and mt2 of *Raillietina* sp. **C** The proportion of synonymous (dS) and non-synonymous (dN) mutations in PCGs

A comparison between the mt genomes of mt1 and mt2 was shown in Table 1. The sequence of mt1 and mt2 shared a nucleotide identity of 95.6% and had the same gene structure and arrangement (Fig. 2A). The length differences between mt1 and mt2 were due to the deletion of 2 nucleotides and the insertion of 12 nucleotides in mt2 of *Raillietina* sp. (Additional file 1: Table S5). The entire mt genome of *Raillietina* sp. contained 12 PCGs (*cox1-3*, *nad1-6*, *nad4L*, *cytb,* and *atp6*), 22 transfer RNAs, 2 ribosomal RNAs, and 2 non-coding regions. The mt genome of *Raillietina* sp. lacked the *atp8* gene, which was identical to known cestode species. The gene order and orientation in mt1 and mt2 of *Raillietina* sp. were transcribed in the same orientation (clockwise direction) (Fig. 2A), which was identical to *Raillietina tetragona*.

**Table 1:** Comparison of mt genome position, length, codons, and anticodon usage in the *Raillietina* sp. mt1 and mt2

Gene	Position	Length		Codon		Anticodon
	Start to End	No nt	No a.a	Start	Stop	
*cox1*	1-1644/1-1644	1644/1644	548/548	ATG/ATG	TAA/TAA	
*trnT*	1630-1694/1630-1694	65/65				TGT/TGT
*rrnL*	1695-2672/1695- 2672	978/978				
*trnC*	2673-2742/2672-2741	70/70				GCA/GCA
*rrnS*	2743-3472/2742-3471	730/730				
*cox2*	3473-4042/3472-4041	570/570	190/190	ATG/ATG	TAA/TAA	
*trnE*	4052-4115/4051-4114	64/64				TTC/TTC
*nad6*	4119-4577/4118-4576	459/459	153/153	ATG/ATG	TAG/TAG	
*trnY*	4580-4646/4579-4645	67/67				GTA/GTA
NCR1	4647-4830/4646-4829	184/184				
*trnS2*	4831-4901/4830-4900	71/71				TGA/TGA
*trnL1*	4903-4976/4902-4975	74/74				TAG/TAG
*trnL2*	5002-5066/5001-5065	65/65				TAA/TAA
*trnR*	5129-5188/5128-5187	60/60				ACG/ACG
*nad5*	5248-6822/5248-6822	1575/1575	525/525	ATG/ATG	TAA/TAA	
NCR2	6823-7411/6823-7411	589/589				
*trnG*	7412-7481/7412-7481	70/70				TCC/TCC
*cox3*	7498-8142/7499-8143	645/645	215/215	ATG/ATG	TAG/TAG	
*trnH*	8152-8225/8153-8226	74/74				GTG/GTG
*cytb*	8229-9341/8230-9342	1113/1113	371/371	ATG/ATG	TAG/TAG	
*nad4L*	9334-9594/9335-9595	261/261	87/87	ATG/ATG	TAG/TAG	
*nad4*	9561-10814/9562-10815	1254/1254	418/418	ATG/ATG	TAG/TAA	
*trnQ*	10816-10879/10817-10880	64/64				TTG/TTG
*trnF*	10885-10947/10886-10948	63/63				GAA/GAA
*trnM*	10945-11013/10946-11014	69/69				CAT/CAT
*atp6*	11020-11526/11021-11527	507/507	169/169	ATG/ATG	TAA/TAA	
*nad2*	11530-12408/11531-12409	879/879	293/293	ATG/ATG	TAG/TAG	
*trnV*	12413-12476/12414-12477	64/64				TAC/TAC
*trnA*	12499-12567/12507-12575	69/69				TGC/TGC
*trnD*	12573-12640/12582-12649	68/68				GTC/GTC
*nad1*	12650-13540/12659-13549	891/891	297/297	ATG/ATG	TAA/TAA	
*trnN*	13565-13625/13574-13634	61/61				GTT/GTT
*trnP*	13639-13704/13648-13713	66/66				TGG/TGG
*trnI*	13705-13770/13714-13779	66/66				GAT/GAT
*trnK*	13781-13842/13790-13851	62/62				CTT/CTT
*nad3*	13853-14200/13862-14209	348/348	116/116	ATG/ATG	TAA/TAA	
*trnS1*	14200-14259/14209-14268	60/60				GCT/GCT
*trnW*	14263-14325/14273-14335	63/63				TCA/TCA

*a.a* amino acids

The complete mt genome of mt1 and mt2 was further compared with the related species of *R. tetragona* from the family Davaineidae and *Hymenolepis diminuta* from the family Hymenolepididae in the order Cyclophyllidea. The genome of mt1 had 74.5% and 71.7% nucleotide identity to *R. tetragona* and *Hymenolepis diminuta*, respectively (Table 2). The complete mtDNA of mt1 was composed of *A* = 25.3%, *T* = 45.1%, *G* = 21.5%, and *C* = 8.1%, while mt2 was composed of *A* = 25.3%, *T* = 45.0%, *G* = 21.5%, and *C* = 8.2%. The AT contents of PCGs in mt1 ranged from 68.4% (*cox*2) to 76.4% (*nad*3), while the AT contents of PCGs in mt2 ranged from 68.1% (*cox*2) to 75.9% (*nad*3). The entire AT contents of mt1 and mt2 in *Raillietina* sp. were 70.4% and 70.3%, respectively (Additional file 1: Table S6). Thus, the sequences of both mtDNAs were mostly favored towards T base, but a minor proportion of C base was detected. The entire mtDNA skew value of *Raillietina* sp. was calculated using the following formulas: AT skew ((*A*–*T*)/(*A* + *T*)) and GC skew ((*G*–*C*)/(*G* + *C*)) [18]. The base compositions of the entire mtDNA of *Raillietina* sp. were inclined toward AT bases in mt1 and mt2 with identical values (AT skew = − 0.3, GC skew = 0.5) (Additional file 1: Table S6).

**Table 2:** Comparison of *Raillietina* sp. mt1 and mt2 sequences with *Raillietina tetragona* and *Hymenolepis diminuta* based on nucleotide sequence identity (%) of PCGs, NCR, and rRNA

Gene	Gene length (bp)				Sequence identity (%)		
	mt1* 14331	mt2 14341	RT 14444	HD 13708	mt1*/mt2	mt1*/RT	mt1*/HD
*cox1*	1644	1644	1617	1593	95.8	81.7	75.1
*cox2*	570	570	573	579	96.3	79.7	71.3
*nad6*	459	459	456	459	93.2	70.5	62.7
*nad5*	1575	1575	1581	1575	94.9	71.0	64.0
*cox3*	645	645	648	651	95.6	74.3	62.3
*cytb*	1113	1113	1092	1098	95.2	79.8	67.2
*nad4L*	261	261	270	261	95.0	70.7	72.7
*nad4*	1254	1254	1251	1248	93.7	75.3	65.0
*atp6*	507	507	507	516	95.4	74.1	65.6
*nad2*	879	879	738	882	96.1	66.6	67.9
*nad1*	891	891	891	891	94.3	78.1	74.4
*nad3*	348	348	348	348	97.1	79.3	68.3
NCR1	184	184	178	185	94.5	31.6	19.5
NCR2	589	589	728	261	100.0	27.6	16.1
*rrnL*	978	978	971	967	96.9	79.3	66.8
*rrnS*	730	730	699	715	97.5	80.2	73.8
Total identity					**95.6**	**74.5**	**71.7**

*Raillietina tetragona* (RT); *Hymenolepis diminuta* (HD); * mt1 was the reference

Sequence variations of mt1 and mt2 were identified in *Raillietina* sp. The heteroplasmic sites in mt1 and mt2 were found in NCR, PCGs, tRNA, rRNA, and intergenes. Most heteroplasmic sites was detected in PCGs, reaching to 79.0% in total sites (Fig. 2B). This heteroplasmic sites of PCGs was mostly the synonymous (dS) mutation (78.4%) (Fig. 2C), and only 21.6% of mutant sites lead to the differences of protein sequences. Thus, the heteroplasmic sites of mt1 and mt2 was mainly distributed in PCG regions.

### Protein coding genes (PCGs) and codon usage

The 12 PCGs of mt1 and mt2 of *Raillietina* sp. encoded 3382 amino acids. The total length of the PCGs in both mts was 10,146 bp, which accounts for 75.5% of the entire mt genome of *Raillietina* sp. The *cox1* and *nad5* genes were longer than other genes, whereas *nad4L* was the shortest gene in the PCGs of two mtDNAs. The initiation and termination codons of PCGs in both mtDNAs were shown in Table 1. The same start codon of ATG was used throughout the PCGs of mt1 and mt2. In mt1, TAA was utilized as a stop codon in six genes of *cox1*, *cox2*, *nad1*, *nad3*, *nad5*, and *atp6*, while TAG was employed in the six genes of *nad6*, *cox3*, *cytb*, *nad2, nad4*, and *nad4L*. In mt2, TAA was used as a stop codon in seven genes of *cox1*, *cox2*, *nad1*, *nad3, nad4*, *nad5*, and *atp6*, whereas TAG was used as a stop codon in five genes of *nad6*, *cox3*, *cytb*, *nad2*, and *nad4L*. Thus, the differences in stop codon usage were noticed only in the *nad4* gene of mt1 (TAG) and mt2 (TAA). The nucleotide sequence identity of 12 PCGs between mt1 and mt2 ranged from 93.2% (*nad6*) to 97.1% (*nad3*). In PCGs, mt1 had the highest nucleotide identity with *R. tetragona* in *cox1* (81.7%) and *cytb* (79.8%) and with *H. diminuta* in *cox1* (75.1%) and *nad1* (74.4%) (Table 2).

**Fig. 3:**
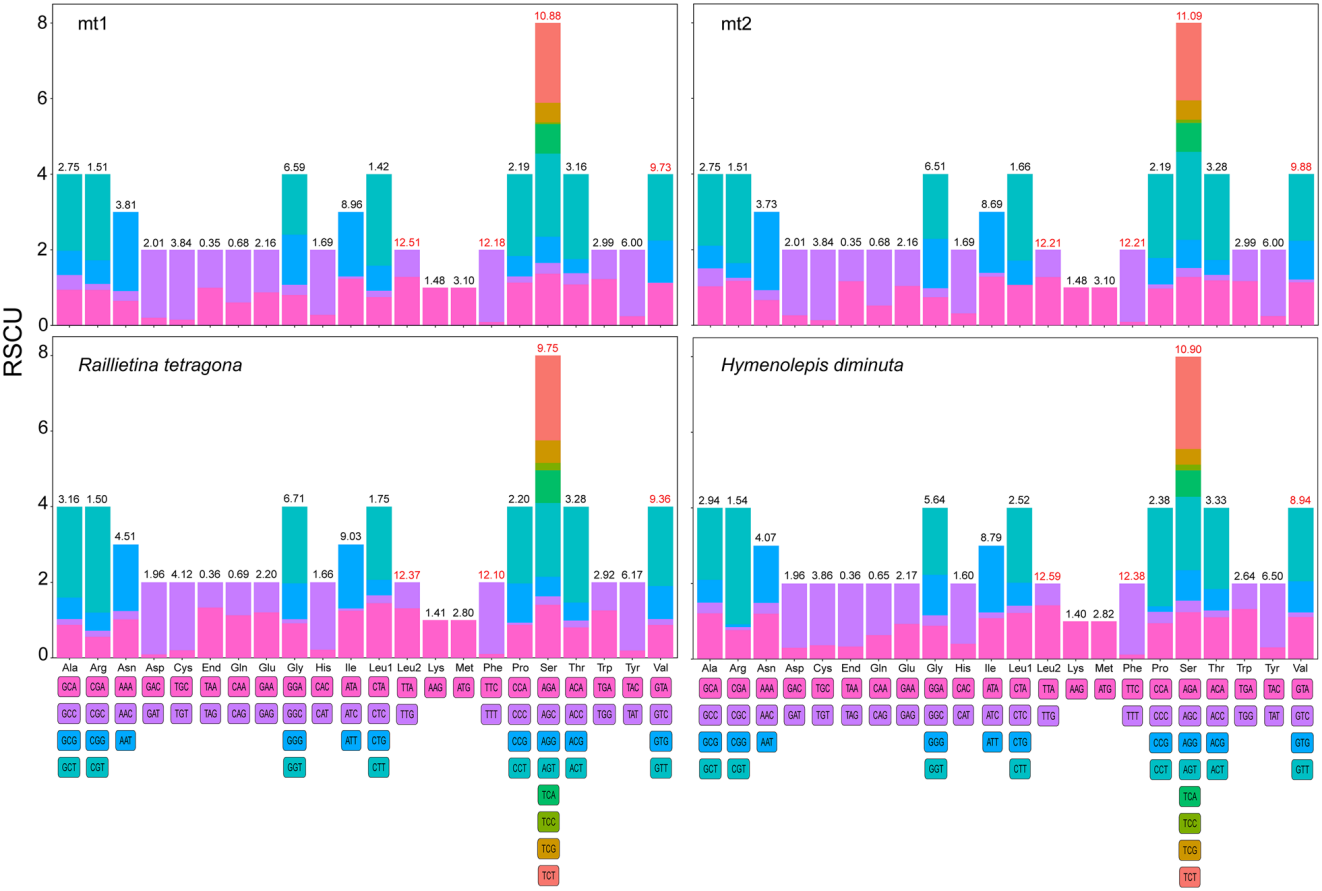
Relative synonymous codon usage (RSCU) of 12 PCGs in the mt1 and mt2 of *Raillietina* sp. compared to *Raillietina tetragona* and *Hymenolepis diminuta*. The type of codon family was indicated on the X-axis, and the percent of amino acid usage was indicated on the top of the bars

The codon usage in the mtDNA of *Raillietina* sp. and *R. tetragona* was analyzed. Relative synonymous codon usage (RSCU) and codon usage associated with amino acids in mt1 and mt2 were shown in Fig. 3. In total, 64 codons were used in the PCGs of mt1 and mt2. Of these, the most frequently utilized amino acids in mt1, mt2, *R. tetragona*, and *H. diminuta* were phenylalanine (TTT) and leucine (TTA). In contrast, the lowest frequency of codon usage was different in mt1 (valine = GTC), mt2 (leucine = CTC), *R. tetragona* (proline = CCC), and *H. diminuta* (arginine = CGC, CGG). However, leucine (CTC = 0%) was not used in mt2 of *Raillietina* sp. RSCU in mt1 and mt2 was almost similar, but slight differences were observed in the total percentage of amino acid usage. All amino acids of mt1, mt2, *R. tetragona*, and *H. diminuta* had more than one codon preference to utilize, except for lysine (AAG) and methionine (ATG), which possess only one codon and lack codon preference (Fig. 3).

### Ribosomal RNA genes (rRNA) and transfer RNA (tRNA)

Two ribosomal RNAs (*rrnL* and *rrnS*) were found in *Raillietina* sp. In mt1 and mt2, *rrnL* was positioned between *trnT* and *trnC*, while *rrnS* was found between *trnC* and *cox2*. The complete length of rRNA in mt1 and mt2 was 1708 bp for each, with an A + T content of 69.8% and 69.3%, respectively. The lengths of *rrnL* (978 bp) and *rrnS* (730 bp) in mt1 and mt2 were identical. *rrnS* had a nucleotide identity of 97.5% between mt1 and mt2, whereas *rrnL* had a nucleotide identity of 96.9%. The *rrnL* and *rrnS* of mt1 had 79.3% and 80.2% nucleotide identities with *rrnL* and *rrnS* of *R. tetragona,* respectively (Table 2).

In mt1 and mt2, different nucleotide lengths were found in 22 tRNAs, ranging from 60 bp (*trnS1*) to 74 bp (*trnH*, *trnL1*). The secondary structures of tRNA in *Raillietina* sp. mt1 and mt2 were predicted (Additional file 1: Fig. S6). The secondary structures of 20 tRNAs in mt1 and mt2 had conventional cloverleaf structures, but *trnS1* and *trnR* lacked a dihydrouridine (DHU) arm. The secondary structures of 20 tRNAs in mt1 and mt2 were composed of DHU arms (2–4 base pairs) and DHU loops (3–11 bases). All 22 tRNA secondary structures of mt1 and mt2 consisted of TψC arm (2–6 base pairs) and TψC loop (3–10 bases). Thus, the secondary structures of tRNA in *Raillietina* sp. were almost similar to those of *R. tetragona*.

### Non-coding region (NCR)

The *Raillietina* sp. mt consisted of NCR1 and NCR2. The length of the NCR in both mt1 and mt2 was 773 bp. The length of NCR2 in mt1 and mt2 (589 bp) was significantly longer than that of NCR1 (184 bp), which was similar to *R. tetragona* (178 bp of NCR1 and 728 bp of NCR2), but was slightly different from *H. diminuta* (185 bp of NCR1 and 261 bp of NCR2) (Table 2). The NCR1 of mt1 and mt2 was located between *trnY* and *trnS2*, whereas NCR2 was located between *nad5* and *trnG*. The total A + T content of NCR in mt1 and mt2 was 73.0% and 72.7%, respectively. The nucleotide sequence identity of mt1 and mt2 on the basis of NCR1 and NCR2 was 94.5% and 100%, respectively (Table 2). The sequence identity of NCR1 and NCR2 in *Raillietina* sp. to *R. tetragona* (31.6% and 27.6%) was higher than that to *H. diminuta* (19.5% and 16.1%), respectively (Table 2). Thus, the different length of NCR resulted in different total lengths of mtDNA, and the low sequence identity of NCR was noticed, which might be the candidate region for molecular identification of species.

The putative secondary structures of NCR1 and NCR2 in mt1, mt2, and *R. tetragona* were compared (Additional file 1: Fig. S7). The potential secondary structure of NCR1 in mt1 was folded into three stem-loop structures, whereas NCR1 in mt2 was folded into four stem-loop structures with many stem and loop sub-structures. Similarly, the potential secondary structures of NCR1 in *R. tetragona* could be folded into two stem-loop structures; one was relatively long with many stems and loops, and the other was short with few stems and loops. However, we could not find any repeat sequences in NCR1 of mt1, mt2, and *R. tetragona*.

The putative secondary structures of NCR2 in both mt1 and mt2 could be folded as a single stem-loop structure and composed of a stem (5 nucleotide pairs) and a loop (5 nucleotides). Likewise, the putative secondary structure of NCR2 in *R. tetragona* was folded into a single stem-loop structure consisting of a stem (13 conical nucleotide pairs) and a loop (3 nucleotides). Some repeat sequences were detected only in NCR2 of mt1, mt2, and *R. tetragona*. The complete length of the repeat sequences in mt1 and mt2 contained 18 identical repeats with 32-nt sequences. In the case of *R. tetragona*, 21 identical repeats of a 34-nt sequences were found (Additional file 1: Fig. S7).

### Phylogenetic analysis based on PCGs

**Fig. 4:**
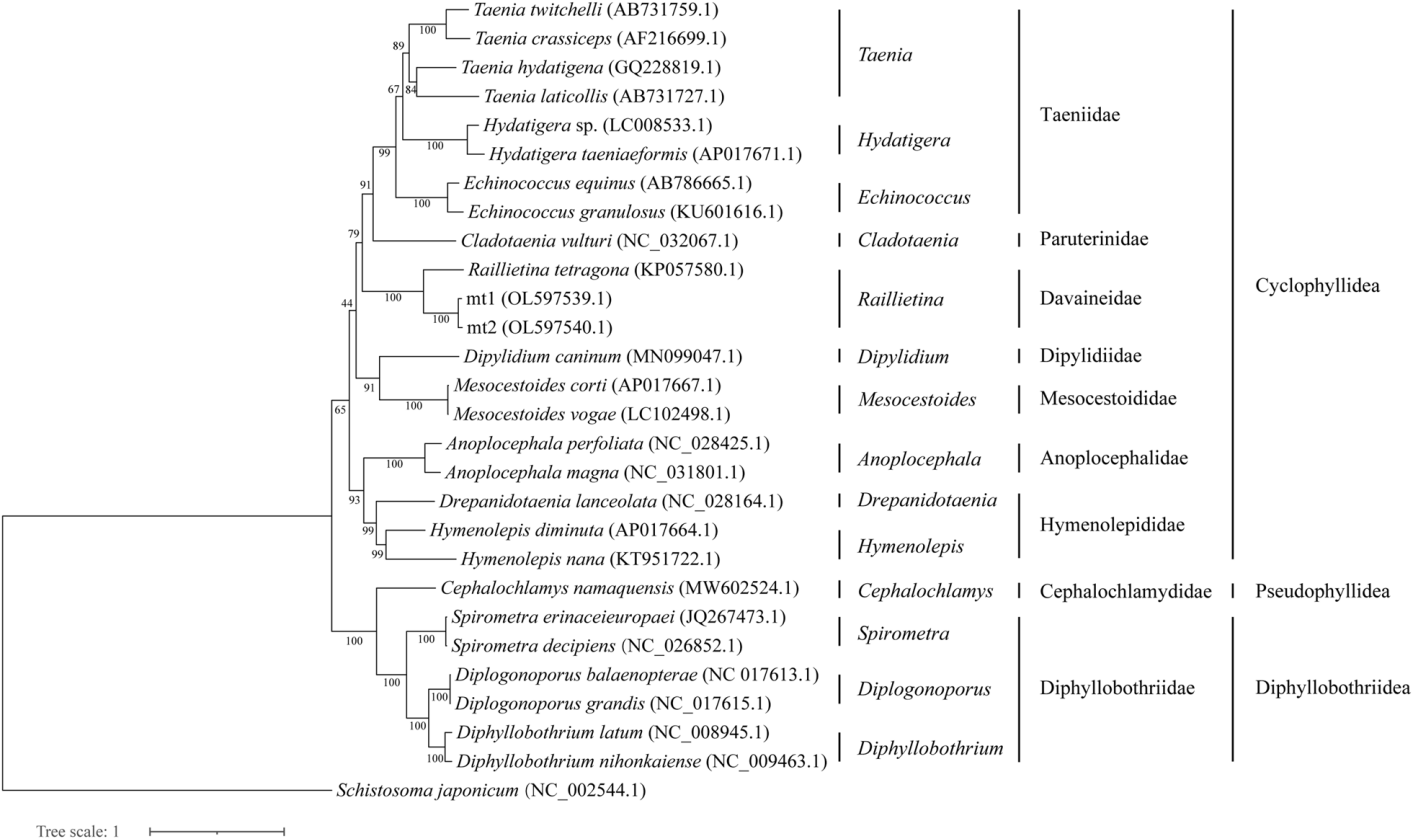
Phylogenetic relationships of some species in the order Cyclophyllidea, Pseudophyllidea, Diphyllobothriidea, and *Raillietina* sp. according to 12 PCGs sequences. Phylogenetic tree was reconstructed based on ML methods. The scale bar represented the number of nucleotide substitutions per site

A maximum likelihood (ML) tree of PCGs from mt1 and mt2 of *Raillietina* sp. and 25 cestode species and one trematode (outgroup) was constructed to analyze the phylogenetic relationships of mt1 and mt2 and cestode species from 14 genera. The mt1 and mt2 were clustered with *R. tetragona* in the Daivaineidae family with strong nodular support (Fig. 4). The mt1 and mt2 sequences were found to be more closely related to *R. tetragona* than to the species from genera *Taenia*, *Hydatigera, Echinococcus*, *Cladotaenia*, *Dipylidium*, *Mesocestoides*, *Anoplocephala*, *Drepanidotaenia*, and *Hymenolepis* in the order Cyclophyllidea. However, *Raillietina* sp. was separated from species of Pseudophyllidea. Thus, this *Raillietina* sp. was identified as a novel species in the genus *Raillietina*.

## Discussions

We sequenced the mt genomes of a novel *Raillietina* sp. derived from wild pangolin using Illumina sequencing and analyzed phylogenetic relationships. Two distinct mitochondrial genomes of mt1 and mt2 were identified in a novel *Raillietina* sp., suggesting the existence of mtDNA heteroplasmy. To the best of our knowledge, this is the first report of mtDNA heteroplasmy in tapeworm parasites. Based on the 18S rRNA and 12 PCGs sequences, the novel *Raillietina* sp. was clustered closely with *Raillietina tetragona* in the family Davaineidea.

The complete mt genomes of mt1 and mt2 were 14,331 bp and 14,341 bp in length, respectively, and had almost the same genome structures and arrangements. The complete mt genome of the *Raillietina* sp. mt1 and mt2 was longer than those of the other Cyclophyllidea and Pseudophyllidea but shorter than that of *R. tetragona* (14,444 bp) [10]. This discrepancy was due to the different lengths of NCR2 and *rrnS*. The mt gene arrangements and the direction of gene transcription in mt1 and mt2 of *Raillietina* sp. were identical to those of most flatworms [10, 19, 20]. However, the gene arrangements of *Raillietina* sp. mt1 and mt2 differed from those of the species in the Taeniidae family in which the positions of *trnL1* and *trnS2* genes were interchanged [7, 21].

In 12 PCGs of *Raillietina* mt1 and mt2, ATG was used as an initiation codon, whereas TAG and TAA were used as termination codons, as in *Raillietina tetragona*, *Cloacotaenia megalops*, and *Hymenolepis nana* [10, 22, 23]. The mt1 and mt2 of *Raillietina* sp. used complete initiation and termination codons, but incomplete codons were not detected, which was consistent with previous findings for some flatworms [10, 20, 22].

The predicted secondary structures of 20 tRNAs in both mts had a conventional cloverleaf structure, but *trnS1* and *trnR* lacked a stable DHU arm, as described in the mtDNA of *Raillietina tetragona* [10]. However, the predicted secondary structures of 18 tRNAs had a typical cloverleaf structure in *H. diminuta*, *H. nana*, and *Cloacotaenia megalops*, except for *trnS1*, *trnS2*, *trnC*, and *trnR* (lacked a DHU arm) [19, 22, 23]. The position of NCR1 in mt1 and mt2 was located between *trnY* and *trnS2*, while NCR2 was found between *nad5* and *trnG*. This position was identical to that of *R. tetragona*, *H. nana*, and *Cladotaenia vulture* [10, 22, 24]; conversely, the position of NCR1 differs from that of taeniid mtDNAs (*trnY* and *tRNL1*).

The secondary structures of NCR1 and NCR2 in both mts were folded into stem-loop structures, which were almost similar to those of *R. tetragona* [10]. Notably, the repeat regions and secondary structure of long NCRs in cestodes had been proposed as control regions to initiate replication and transcription [25, 26], yet this remains unexplored and requires further investigation. The presence of repeat sequences was a distinguishing feature of the cestode NCR [19]. In this study, the repeat sequence was detected only in NCR2 of mt1 and mt2 of *Raillietina* sp., similar to that of *R. tetragona* and *H. diminuta* [10, 22]. However, the size of NCR2 in *Raillietina* sp. mt1 and mt2 was different from those of *R. tetragona*, but was considerably different from *H. diminuta*. Thus, the total differences of mt genome in *Raillietina* sp., *R. tetragona*, and *H. diminuta* are mainly due to the variation in the size of NCR2.

Mitochondrial heterogeneity had been widely reported at the intra- and intercellular levels in humans and animals [27]. In flatworms, evidence of mtDNA heteroplasmy was documented in parasitic trematode of *Schistosoma mansoni* [28]. However, there is a lack of reporting on mtDNA heteroplasmy in individual tapeworm parasites to date. Herein, we identified a variant mtDNA sequence in a single worm from a wild pangolin with little difference in length. In particular, the mtDNA heteroplasmic mutations of coding genes and NCRs were detected mainly in PCGs of *Raillietina* sp. and could be a source of mtDNA sequence heterogeneity.

## Conclusions

We presented the heterogeneity of the mt genome data of *Raillietina* sp. from wild pangolin via Illumina sequencing of total DNA. *Raillietina* sp. was a novel species in the Davaineidae family according to 18S rRNA, *ITS2*, and both mt genomes. This provides insights into the heterogeneity of the mt genome in cestodes.

## Supplementary Information

**Additional file 1**: **Fig. S1**. Morphological observation of worm originated from wild pangolin. **Fig. S2**: Alignment and phylogenetic analysis of *ITS2* region from worms of sample A and sample B. **Fig. S3**: PCR fragments amplified from uncertain regions of the two mitochondrial sequences. **Fig. S4**: Reads mapping result of the mt DNA of *Raillietina* sp. was visualized by IGV. **Fig. S5:** Sequences alignment of mt1 and mt2 of *Raillietina* sp*.* amplified by PCR using two different primers (JB11-JB12 and mt2-PCR). **Fig. S6**: Predicted secondary structure of 22 tRNAs of mt1 and mt2 of *Raillietina* sp. compared with *Raillietina tetragona.* **Fig. S7**: Putative secondary structure of non-coding regions (NCR1, NCR2) in mt1, mt2, and related species of *R. tetragona* predicted by mfolds. **Table S1**: Primers used for amplification of repeat and N-sequence/unidentified regions of mt1 and mt2 of *Raillietina* sp. **Table S2**: The retrieved 18S rRNA sequences of observed *Raillietina* sp. and species in the Cyclophyllidea, Diphyllobothriidae, and Schistosomatidae (Trematode as outgroup) for phylogenetic analysis. **Table S3**: The *ITS2* sequences of cestode parasites retrieved from GenBank and sequences from two samples of current *Raillietina* sp. **Table S4**: The mt1 and mt2 of *Raillietina* sp. and the downloaded mt genome of 12 PCGs from the order of Cyclophyllidea, Pseudophyllidea, Diphyllobothriidae, and Schistosomatidae (outgroup). **Table S5**: The number of nucleotide deletions and insertions observed between mt1 and mt2 of *Raillietina* sp. **Table S6**: Nucleotide compositions (%) of PCGs, entire mt genome, transfer RNA, ribosomal RNA, and skew value of mt1 and mt2 of *Raillietina* sp*.*

## Abbreviations

WGA: Whole genome amplification
PCR: Polymerase chain reaction
MtDNA: Mitochondria DNA
PCG: Protein coding gene
RSCU: Relative synonymous codon usage
tRNA: Transfer RNA
NCR: Non coding region
