# Supplementary material for: Heteroplasmic mitochondrial genomes of a Raillietina tapeworm in wild Pangolin
Source: Parasit Vectors. 2022 Jun 13;15:204. doi: 10.1186/s13071-022-05301-y (PMC9195439; doi:10.1186/s13071-022-05301-y)
Supplement: Supplementary file 1 — Additional file 1: Fig. S1. Morphological observation of worm originated from wild pangolin. Fig. S2: Alignment and phylogenetic analysis of ITS2 region from worms of sample A and sample B. Fig. S3: PCR fragments amplified from uncertain regions of the two mitochondrial sequences. Fig. S4: Reads mapping result of the mt DNA of Raillietina sp. was visualized by IGV. Fig. S5: Sequences alignment of mt1 and mt2 of Raillietina sp. amplified by PCR using two different primers (JB11-JB12 and mt2-PCR). Fig. S6: Predicted secondary structure of 22 tRNAs of mt1 and mt2 of Raillietina sp. compared with Raillietina tetragona. Fig. S7: Putative secondary structure of non-coding regions (NCR1, NCR2) in mt1, mt2, and related species of R. tetragona predicted by mfolds. Table S1: Primers used for amplification of repeat and N-sequence/unidentified regions of mt1 and mt2 of Raillietina sp. Table S2: The retrieved 18S rRNA sequences of observed Raillietina sp. and species in the Cyclophyllidea, Diphyllobothriidae, and Schistosomatidae (Trematode as outgroup) for phylogenetic analysis. Table S3: The ITS2 sequences of cestode parasites retrieved from GenBank and sequences from two samples of current Raillietina sp. Table S4: The mt1 and mt2 of Raillietina sp. and the downloaded mt genome of 12 PCGs from the order of Cyclophyllidea, Pseudophyllidea, Diphyllobothriidae, and Schistosomatidae (outgroup). Table S5: The number of nucleotide deletions and insertions observed between mt1 and mt2 of Raillietina sp. Table S6: Nucleotide compositions (%) of PCGs, entire mt genome, transfer RNA, ribosomal RNA, and skew value of mt1 and mt2 of Raillietina sp. [file 13071_2022_5301_MOESM1_ESM.docx]

**Supplementary Figures and Tables:**


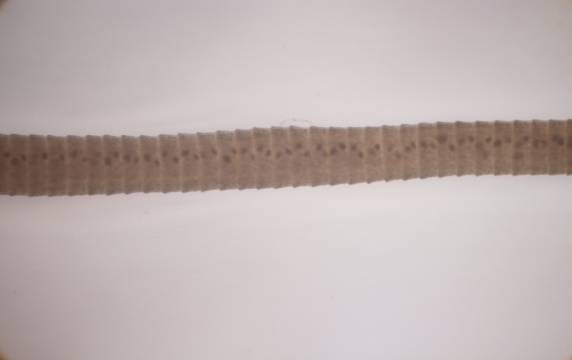


**Additional file1:** **Fig. S1.** Morphological observation of worm originated from wild pangolin


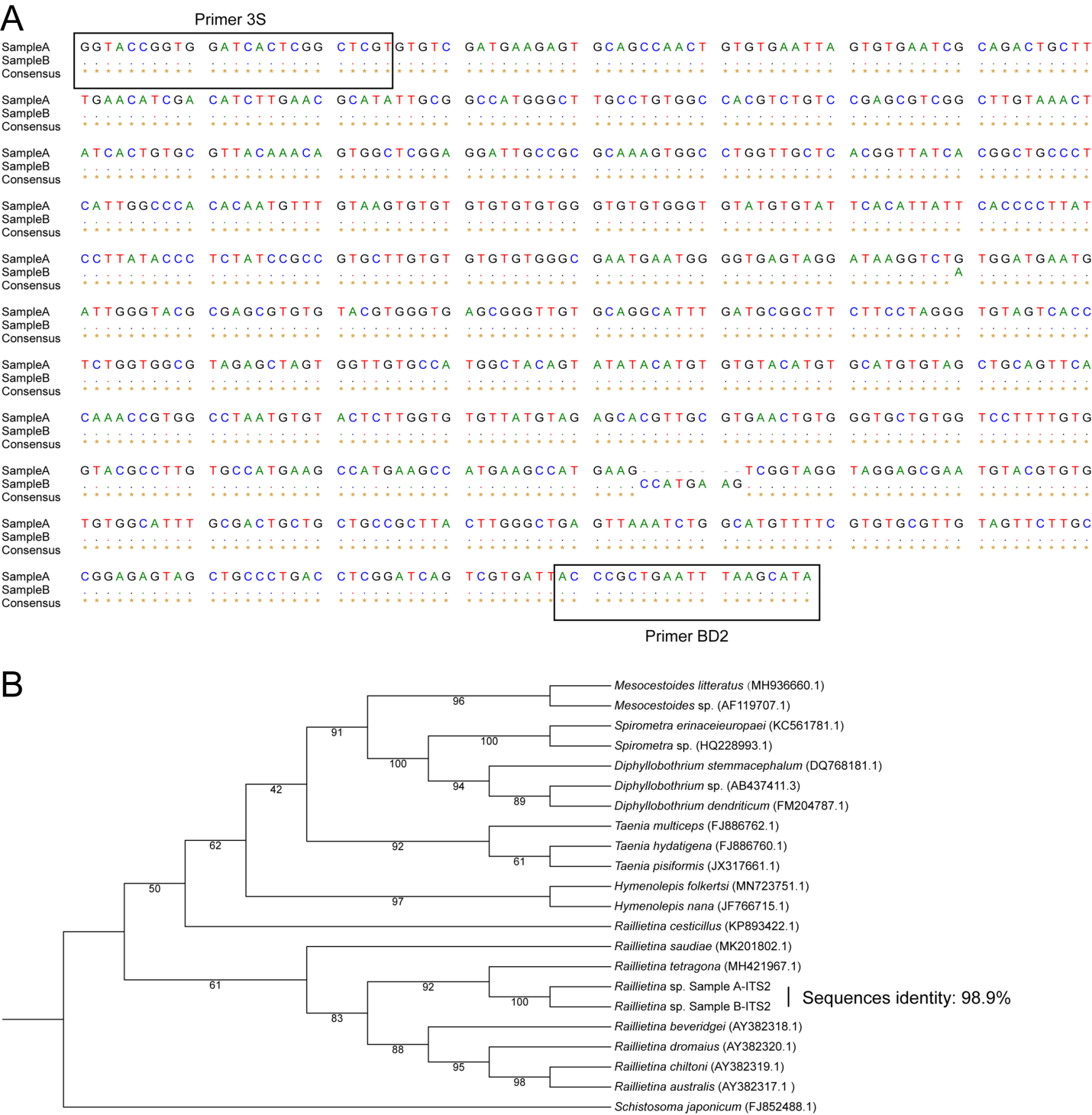


**Additional file1:** **Fig. S2.** Alignment and phylogenetic analysis of *ITS2* region from worms of sample A and sample B.

**
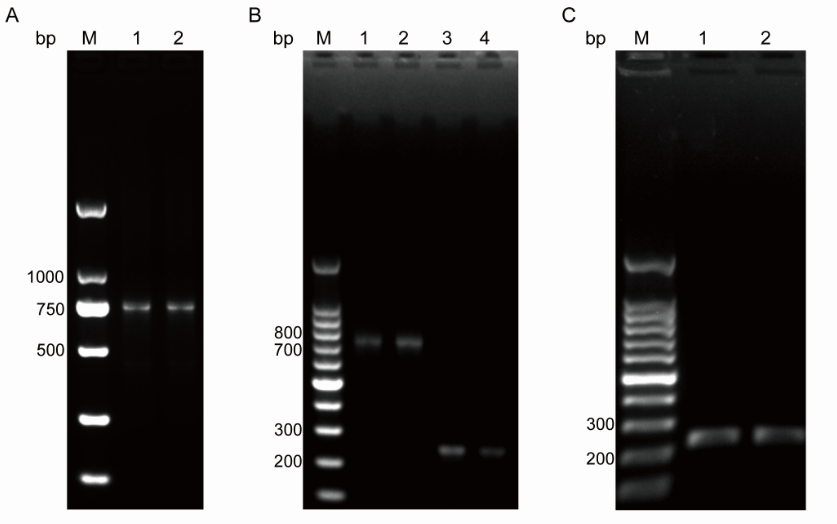
**

**Additional file1:** **Fig. S3.** PCR fragments amplified from uncertain regions of the two mitochondrial sequences. A) lanes 1-2, mt1 repeat sequences; B) lanes 1-2, mt2 repeat sequences; lanes 3-4, mt1 N sequences; C) lanes 1-2, mt2 N-sequences; lanes M: DNA marker.


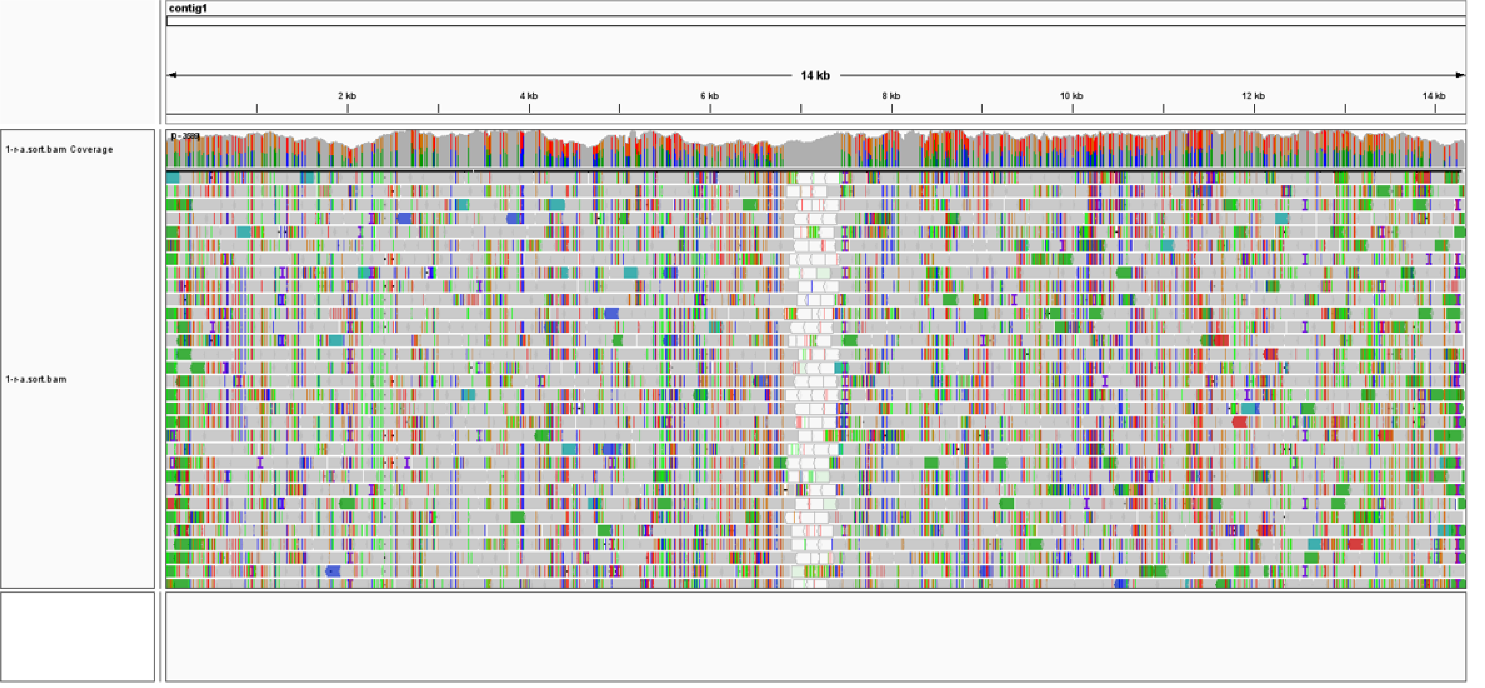


**Additional file1:** **Fig. S4.** Reads mapping result of the mt DNA of *Raillietina* sp. was visualized by IGV. The result shows that each base account for half of the mutation site.

**
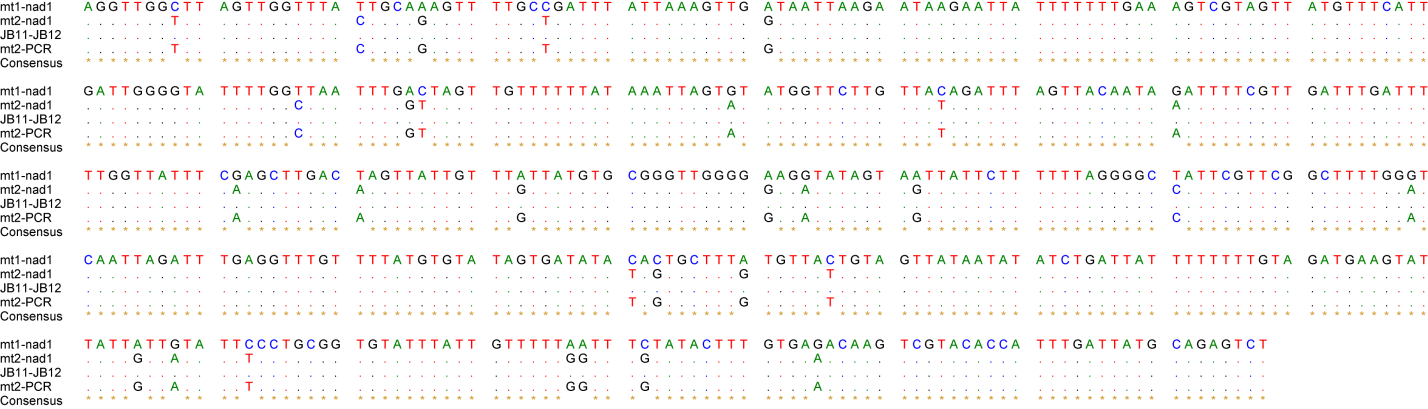
**

**Additional file1:** **Fig. S5.** Sequences alignment of mt1 and mt2 of *Raillietina* sp*.* amplified by PCR using two different primers (JB11-JB12 and mt2-PCR).


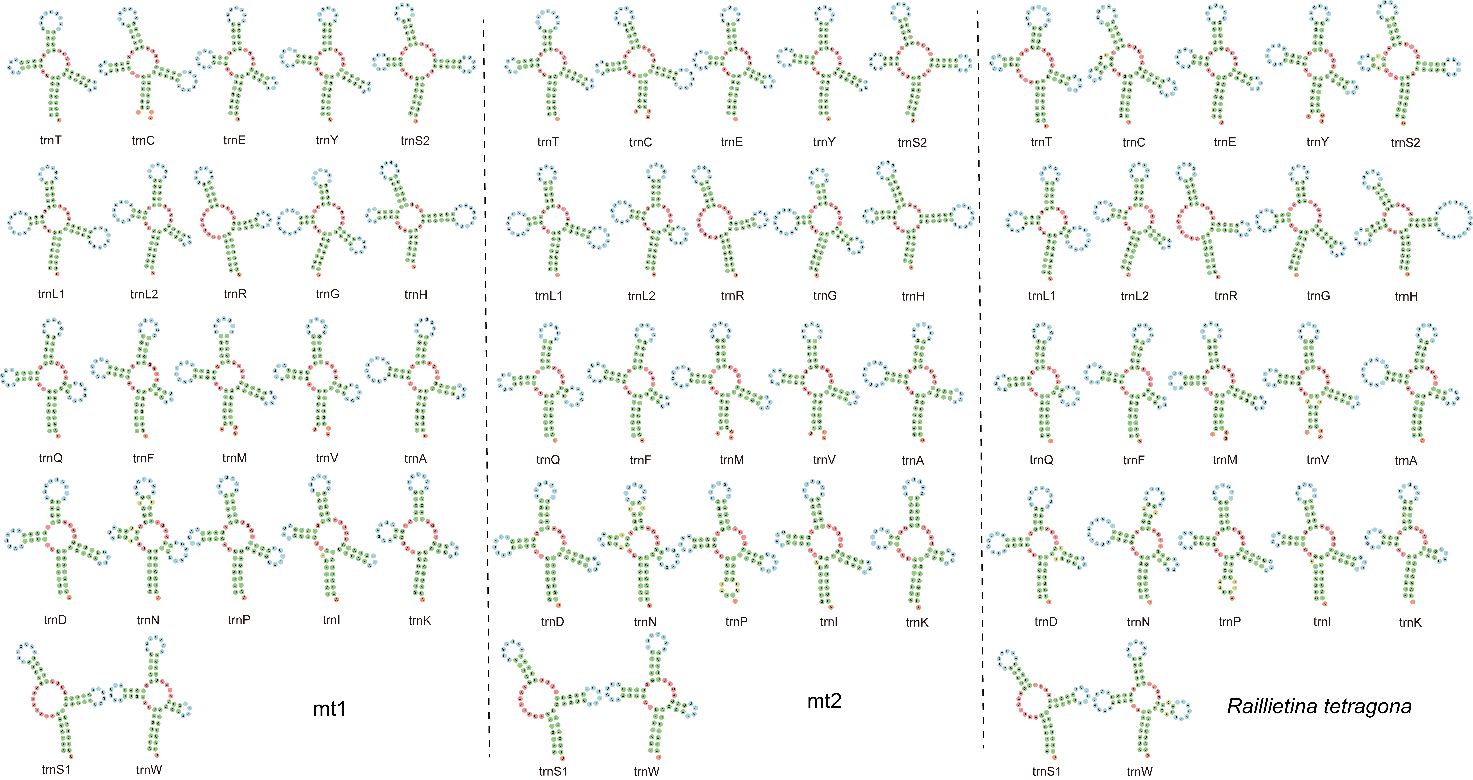


**Additional file1:** **Fig. S6.** Predicted secondary structure of 22 tRNAs in mt1 and mt2 of *Raillietina* sp. compared with *Raillietina tetragona.*


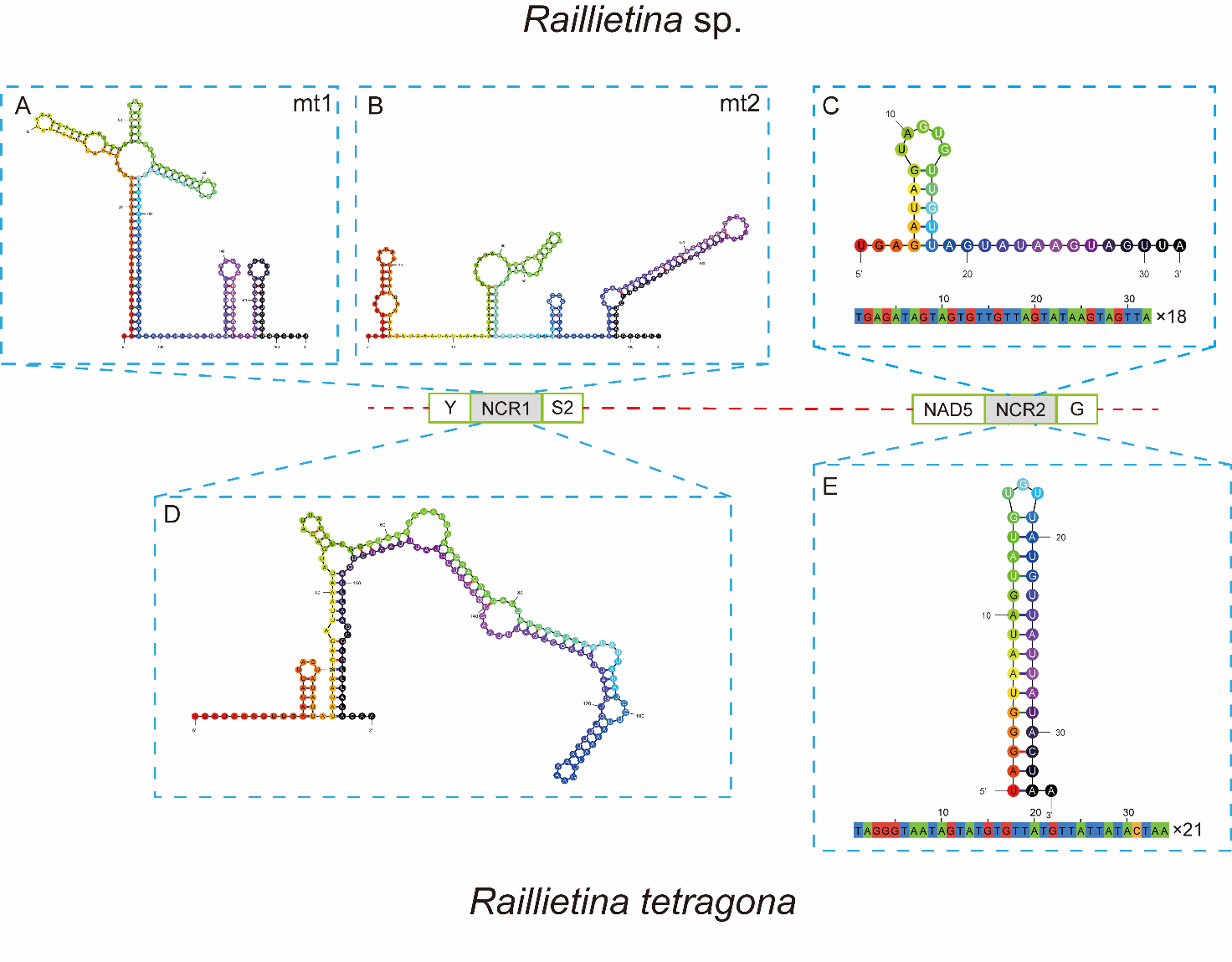


**Additional file1:** **Fig. S7.** Putative secondary structure of non-coding regions (NCR1, NCR2) in mt1, mt2, and related species of *R. tetragona* predicted by mfolds. A) non-repetitive region of NCR1 in mt1; B) non-repetitive region of NCR1 in mt2; C) repeat region of NCR2 in both mts; D) NCR1 of *R. tetragona*; E) repeat region of NCR2 in *R. tetragona.* NCR1 was found between *trnY* and *trnS2*; NCR2 was found between *nad5* and *trnG*.

**Additional file1: Table S1.** Primers used for amplification of repeat and N-sequence / unidentified regions of mt1 and mt2 of *Raillietina* sp.

| **mtDNA** | **Uncertain regions** | **Forward Primer (5′ to 3′)** | **Reverse Primer (5′ to 3′)** |
| --- | --- | --- | --- |
| mt1 | N sequence | CTTTGCTTTTTTATGAGTATTTGAT | ACACCAACATAACAAAAGTAAAATAA |
|  | Repeat sequence | ACTAAAATTATTAATTTATTCGATGTAT | ATAAAGGTATTACAGACATTCCAAC |
| mt2 | N sequence | TCTGTTTTCTTTGCTTTTTTG | AAGTAAAATAATAGGCGGGTC |
|  | Repeat sequence | TTTATTCGATATTTTTCTTCCTT | GTATTACAGACATCCCAAACCAC |

Note: N- unidentified sequence region

**Additional file1:** **Table S2.** The retrieved 18S rRNA sequences of observed *Raillietina* sp. and species in the Cyclophyllidea, Diphyllobothriidae, and Schistosomatidae (Trematode as outgroup) for phylogenetic analysis.

| **Order** | **Family** | **Species** | **Size (bp)** | **Accession number** |
| --- | --- | --- | --- | --- |
| Cyclophyllidea | Taeniidae | *Taenia saginata* | 2608 | JQ609338.1 |
|  |  | *Taenia asiatica* | 2583 | GQ260088.1 |
|  |  | *Taenia multiceps* | 2574 | GQ260089.1 |
|  |  | *Taenia solium* | 2599 | GQ260091.1 |
|  |  | *Taenia crassiceps* | 2665 | AB731618.1 |
|  |  | *Hydatigera krepkogorski* | 2752 | AB731632.1 |
|  |  | *Hydatigera kamiyai* | 2765 | AB731630.1 |
|  |  | *Hydatigera taneaformis* | 2891 | JQ609340.1 |
|  |  | *Echinococcus granulosus* | 2394 | U27015.1 |
|  |  | *Echinococcus equinus* | 2266 | AB731640.1 |
|  | Mesocestoididae | *Mesocestiodes litteratus* | 2154 | DQ643002.1 |
|  |  | *Mesocestiodes corti* | 2294 | AF286984.1 |
|  | Dipylidiidae | *Diplidium caninum* | 2406 | AB731643.1 |
|  | Hymenolepididae | *Hymenolepis diminuta* | 2174 | AF124475.1 |
|  |  | *Hymenolepis microstoma* | 2140 | AJ287525.1 |
|  |  | *Hymenolepis nana* | 2244 | AY193875.1 |
|  | Davaineidae | *Raillietina* sp*.* | 2183 | EU665467.1 |
|  |  | *Raillietina sonini* | 2183 | EU665468.1 |
|  |  | *Fuhrmannetta malakartis* | 2185 | EU665463.1 |
|  |  | ***Raillietina* sp.** | 2214 | OL547740.1 |
|  |  | *Raillietina* sp. | 2183 | EU665466.1 |
|  |  | *Raillietina dromaius* | 2220 | AY382314.1 |
|  |  | *Raillietina chiltoni* | 2242 | AY382313.1 |
|  |  | *Raillietina australis* | 2235 | AY382311.1 |
|  |  | *Raillietina mitchelli* | 2238 | AY382315.1 |
|  |  | *Raillietina beveridgei* | 2219 | AY382312.1 |
|  |  | *Raillietina australis* | 2238 | AF286980.1 |
| **Diphyllobothriidea** | Diphyllobothriidae | *Spirometra erinacei* | 2182 | D64072.1 |
|  |  | *Spirometra* sp. | 2242 | HQ228992.1 |
|  |  | *Diplogonaporus balaenopterae* | 2022 | KY552792.1 |
|  |  | *Diplogonaporus grandis* | 2156 | AB353272.1 |
|  |  | *Dibothriocephalus nihonkaiensis* | 2171 | AB374225.1 |
|  |  | *Diphyllobothrium latum* | 3356 | KF218246.1 |
| [Strigeidida](https://animaldiversity.org/accounts/Strigeatida/classification/#Strigeatida) | Schistosomatidae | *Schistosoma japonicum* | 1883 | AY157226.1 |

**Additional file1:** **Table S3.** The *ITS2* sequences of cestode parasites retrieved from GenBank and sequences from two samples of current *Raillietina* sp.

| **Order** | **Family** | **Species** | **Size (bp)** | **Accession No** |
| --- | --- | --- | --- | --- |
| Cyclophyllidea | Mesocestoididae | *Mesocestoides* sp*.* | 559 | AF119707.1 |
|  |  | *Mesocestoides litteratus* | 1340 | MH936660.1 |
|  | Taeniidae | *Taenia multiceps* | 1359 | FJ886762.1 |
|  |  | *Taenia hydatigena* | 1259 | FJ886760.1 |
|  |  | *Taenia pisiformis* | 1300 | JX317661.1 |
|  | Hymenolepididae | *Hymenolepis folkertsi* | 833 | MN723751.1 |
|  |  | *Hymenolepis nana* | 1379 | JF766715.1 |
|  | Davaineidae | *Raillietina cesticillus* | 2236 | KP893422.1 |
|  |  | *Raillietina saudiae* | 726 | MK201802.1 |
|  |  | *Raillietina tetragona* | 926 | MH421967.1 |
|  |  | ***Raillietina* sp. sample A** | 850 |  |
|  |  | ***Raillietina* sp. sample B** | 858 |  |
|  |  | *Raillietina beveridgei* | 1081 | AY382318.1 |
|  |  | *Raillietina dromaius* | 973 | AY382320.1 |
|  |  | *Raillietina australis* | 984 | AY382317.1 |
|  |  | *Raillietina beveridgei* | 1081 | AY382318.1 |
| Diphyllobothriidea | Diphyllobothriidae | *Spirometra* sp*.* | 1307 | HQ228993.1 |
|  |  | *Spirometra erinaceieuropaei* | 1307 | KC561781.1 |
|  |  | *Diphyllobothrium stemmacephalum* | 1251 | DQ768181.1 |
|  |  | *Diphyllobothrium* sp*.* | 1238 | AB437411.3 |
|  |  | *Diphyllobothrium dendriticum* | 1323 | FM204787.1 |
| Strigeidida | Schistosomatidae | *Schistosoma japonicum* | 1541 | FJ852488.1 |

**Additional file1:** **Table S4.** The mt1 and mt2 of *Raillietina* sp. and the downloaded mt genome of 12 PCGs from the order of Cyclophyllidea, Pseudophyllidea, Diphyllobothriidae, and Schistosomatidae (out-group).

| **Order** | **Family** | **Species** | **Size (bp)** | **Accession number** |
| --- | --- | --- | --- | --- |
| Cyclophyllidea | Mesocestoididae | *Mesocestoides vogae* | 13892 | LC102498.1 |
|  |  | *Mesocestoides corti* | 13670 | AP017667.1 |
|  |  | *Dipylidium caninum* | 14226 | MN099047.1 |
|  | Taeniidae | *Echinococcus granulosus* | 13579 | KU601616.1 |
|  |  | *Echinococcus equinus* | 13605 | AB786665.1 |
|  |  | *Hydatigera* sp. | 13840 | LC008533.1 |
|  |  | *Hydatigera taeniaeformis* | 13822 | AP017671.1 |
|  |  | *Taenia crassiceps*  *Taenia twitchelli* | 13503  13519 | AF216699.1  AB731759.1 |
|  |  | *Taenia laticollis* | 13483 | AB731727.1 |
|  |  | *Taenia hydatigena* | 13492 | GQ228819.1 |
|  | Hymenolepididae | *Drepanidotaenia lanceolata* | 13573 | NC_028164.1 |
|  |  | *Hymenolepis diminuta* | 13708 | AP017664.1 |
|  |  | *Hymenolepis nana* | 13764 | KT951722.1 |
|  | Davaineidae | *Raillietina tetragona* | 14444 | KP057580.1 |
|  |  | **mt1 of *Raillietina* sp.** | 14331 | OL597539.1 |
|  |  | **mt2 of *Raillietina* sp.** | 14341 | OL597540.1 |
|  | Paruterinidae | *Cladotaenia vulturi* | 13411 | NC_032067.1 |
|  | Anoplocephalidae | *Anoplocephala perfoliata* | 14459 | NC_028425.1 |
|  |  | *Anoplocephala magna* | 13759 | NC_031801.1 |
| Pseudophyllidea | Cephalochlamydidae | *Cephalochlamys namaquensis* | 13696 | MW602524.1 |
| Diphyllobothriidea | Diphyllobothriidae | *Spirometra erinaceieuropaei* | 13641 | JQ267473.1 |
|  |  | *Spirometra decipiens* | 13641 | NC_026852.1 |
|  |  | *Diplogonoporus balaenopterae* | 13724 | NC_017613.1 |
|  |  | *Diplogonoporus grandis* | 13725 | NC_017615.1 |
|  |  | *Diphyllobothrium latum* | 13608 | NC_008945.1 |
|  |  | *Diphyllobothrium nihonkaiense* | 13747 | NC_009463.1 |
| [Strigeidida](https://animaldiversity.org/accounts/Strigeatida/classification/#Strigeatida) | Schistosomatidae | *Schistosoma japonicum* | 14085 | NC_002544.1 |

**Additional file1:** **Table S5.** The number of nucleotide deletions and insertions observed between mt1 and mt2 of *Raillietina* sp*.*

| **mt1 positions** | **mt2** | |
| --- | --- | --- |
|  | **Nucleotide deletion** | **Nucleotide insertion** |
| 2,028-2,029 |  | G |
| 2,429-2,430 | TA |  |
| 5,204-5,205 |  | T |
| 7,493-7,493 |  | T |
| 12,478-12,479 |  | TTGGGGG |
| 12,568-12,569 |  | T |
| 14,256-14,257 |  | T |

**Additional file1:** **Table S6.** Nucleotide compositions (%) of PCGs, entire mt genome, transfer RNA, ribosomal RNA, and skew value of mt1 and mt2 of *Raillietina* sp*.*

| **Gene** | **A %** | **G %** | **C %** | **T %** | **A+T %** | **G+C %** | **AT skew** | **GC skew** |
| --- | --- | --- | --- | --- | --- | --- | --- | --- |
| *cox1* | 23.7/23.9 | 21.6/21.1 | 9.9/9.9 | 44.8/45.1 | 68.6/69.0 | 31.4/31.0 | -0.3/-0.3 | 0.4/0.4 |
| *cox2* | 27.0/26.8 | 22.5/22.8 | 9.1/9.2 | 41.4/41.2 | 68.4/68.1 | 31.6/31.9 | -0.2/-0.2 | 0.4/0.4 |
| *nad6* | 23.3/23.3 | 20.7/21.1 | 7.6/7.4 | 48.4/48.2 | 71.7/71.5 | 28.3/28.5 | -0.4/-0.4 | 0.5/0.5 |
| *nad5* | 25.0/25.2 | 19.5/19.2 | 9.6/9.9 | 45.9/45.7 | 70.9/70.9 | 29.1/29.1 | -0.3/-0.3 | 0.3/0.3 |
| *cox3* | 22.5/21.9 | 22.9/23.2 | 6.7/7.9 | 47.9/47.0 | 70.4/68.8 | 29.6/31.2 | -0.4/-0.4 | 0.6/0.5 |
| *cytb* | 23.1/23.6 | 22.0/21.3 | 9.2/9.4 | 45.7/45.7 | 68.8/69.3 | 31.2/30.7 | -0.3/-0.3 | 0.4/0.4 |
| *nad4L* | 22.6/23.8 | 21.1/20.3 | 7.6/6.9 | 48.7/49.0 | 71.3/72.8 | 28.7/27.2 | -0.4/-0.4 | 0.5/0.5 |
| *nad4* | 23.5/23.4 | 21.9/21.9 | 6.9/7.8 | 47.7/46.9 | 71.2/70.3 | 28.8/29.7 | -0.3/-0.3 | 0.5/0.5 |
| *atp6* | 25.4/25.0 | 19.5/19.9 | 10.3/9.3 | 44.8/45.8 | 70.2/70.8 | 29.8/29.2 | -0.3/-0.3 | 0.3/0.4 |
| *nad2* | 17.9/17.3 | 23.1/23.5 | 5.5/5.5 | 53.5/53.7 | 71.3/71.0 | 28.7/29.0 | -0.5/-0.5 | 0.6/0.6 |
| *nad1* | 22.6/22.5 | 22.6/23.2 | 7.0/6.6 | 47.8/47.7 | 70.4/70.1 | 29.6/29.9 | -0.4/-0.4 | 0.5/0.6 |
| *nad3* | 26.2/25.9 | 20.1/20.4 | 3.4/3.7 | 50.3/50.0 | 76.4/75.9 | 23.6/24.1 | -0.3/-0.3 | 0.7/0.7 |
| Cmt | 25.3/25.3 | 21.5/21.5 | 8.1/8.2 | 45.1/45.0 | 70.4/70.3 | 29.6/29.7 | -0.3/-0.3 | 0.5/0.5 |
| EPCG | 23.4/23.4 | 21.5/21.5 | 8.2/8.3 | 46.9/46.8 | 70.3/70.2 | 29.7/29.8 | -0.3/-0.3 | 0.5/0.4 |
| EtRNA | 29.1/29.8 | 20.9/20.3 | 9.8/9.4 | 40.2/40.5 | 69.3/70.2 | 30.7/29.8 | -0.2/-0.2 | 0.4/0.4 |
| ErRNA | 28.5/28.6 | 20.7/20.8 | 9.5/9.9 | 41.3/40.7 | 69.8/69.3 | 30.2/30.7 | -0.2/-0.2 | 0.4/0.4 |
| ENCR | 33.4/33.4 | 25.5/25.5 | 1.6/1.8 | 39.5/39.3 | 73.0/72.7 | 27.0/27.3 | -0.1/-0.1 | 0.9/0.9 |

Complete mitochondrial (Cmt), Entire (PCG, tRNA, rRNA, NCR).
